# Performance of Novel GPT-4 in Otolaryngology Knowledge Assessment

**DOI:** 10.1007/s12070-024-04935-x

**Published:** 2024-08-03

**Authors:** Lucy Revercomb, Aman M. Patel, Daniel Fu, Andrey Filimonov

**Affiliations:** Department of Otolaryngology – Head and Neck Surgery, Rutgers New Jersey Medical School, 185 S Orange Ave Newark, Newark, NJ 07103 USA

**Keywords:** GPT-4, Otolaryngology, Artificial intelligence, Board examination, Medical education

## Abstract

**Purpose:**

GPT-4, recently released by OpenAI, improves upon GPT-3.5 with increased reliability and expanded capabilities, including user-specified, customizable GPT-4 models. This study aims to investigate updates in GPT-4 performance vs. GPT-3.5 on Otolaryngology board-style questions.

**Methods:**

150 Otolaryngology board-style questions were obtained from the BoardVitals question bank. These questions, which were previously assessed with GPT-3.5, were inputted into standard GPT-4 and a custom GPT-4 model designed to specialize in Otolaryngology board-style questions, emphasize precision, and provide evidence-based explanations.

**Results:**

Standard GPT-4 correctly answered 72.0% and custom GPT-4 correctly answered 81.3% of the questions, vs. GPT-3.5 which answered 51.3% of the same questions correctly. On multivariable analysis, custom GPT-4 had higher odds of correctly answering questions than standard GPT-4 (adjusted odds ratio 2.19, *P* = 0.015). Both GPT-4 and custom GPT-4 demonstrated a decrease in performance between questions rated as easy and hard (*P* < 0.001).

**Conclusions:**

Our study suggests that GPT-4 has higher accuracy than GPT-3.5 in answering Otolaryngology board-style questions. Our custom GPT-4 model demonstrated higher accuracy than standard GPT-4, potentially as a result of its instructions to specialize in Otolaryngology board-style questions, select exactly one answer, and emphasize precision. This demonstrates custom models may further enhance utilization of ChatGPT in medical education.

## Introduction

As artificial intelligence (AI) continues to improve, AI models are being rapidly introduced and integrated into medicine. Recent studies have demonstrated potential benefit of ChatGPT in medical education, including our previous study which found that ChatGPT was able to answer 51% of 150 Otolaryngology board-style questions correctly [1, 2]. In March 2023, OpenAI released GPT-4, improving ChatGPT with increased reliability and expanded capabilities, including the ability to design user-specified, customized models. This study assesses the performance of standard and customized GPT-4 models compared to previous ChatGPT versions on 150 Otolaryngology board-style questions.

## Methods

The same 150 Otolaryngology board-style questions utilized in our previous study of GPT-3.5 were obtained from BoardVitals (https://www.boardvitals.com/*)*, across ten topics and three difficulty levels [2]. These questions were inputted into standard GPT-4 (https://openai.com/gpt-4*)* and a custom GPT-4 model (https://chat.openai.com/g/g-PyDG5N7Ko-ent-expert-mcq-solver*).* Using the built-in model customization interface, the custom GPT-4 model was instructed to specialize in Otolaryngology board-style questions, emphasizing precision, selecting one answer, providing evidence-based explanations, and validating answers using the internet. The independent samples *t*-test and multivariable binary logistic regression were implemented with SPSS version 25 (IBM).

## Results

Of the 150 board-style questions assessed, standard GPT-4 answered 108 (72.0%) and custom GPT-4 answered 122 (81.3%) correctly (Table 1). Both standard (90.0% vs. 46.0%) and custom GPT-4 (98.0% vs. 62.0%) demonstrated a decrease in performance between “easy” and “hard” questions (*P* < 0.001). For 111 (74.0%) and 113 (73.5%) questions, respectively, standard and custom GPT-4 selected the most common answer option chosen by Otolaryngology trainees. On multivariable analysis adjusting for subject, difficulty, question length, answer length, percent trainees correct, standard vs. custom GPT-4, and GPT-4 response length, custom GPT-4 (adjusted odds ratio [aOR] 2.19, 95% confidence interval [CI] 1.16–4.11, *P* = 0.015) and plastic and reconstructive subject (aOR 7.41, 95% CI 1.44–38.05, *P* = 0.016) remained associated with GPT-4 answering correctly.

**Table 1:** Characteristics of 150 multiple-choice questions

Subject	Question Length, mean characters (SD)	Trainees Correct, mean (SD)	Correct Answer Option Length, mean characters (SD)	GPT-4 Correct Response, *n* (%)	GPT-4 Response Length, mean characters (SD)	Custom GPT-4 Correct Response, *n* (%)	Custom GPT-4 Response Length, mean characters (SD)
Allergy	253.1 (139.9)	77.3 (13.1)	23.0 (15.9)	9 (60.0)	1242.9 (515.6)	11 (73.3)	1404.6 (453.4)
Endocrine	149.3 (149.7)	78.0 (13.6)	49.1 (45.2)	12 (80.0)	1148.6 (343.2)	11 (73.3)	1494.8 (248.3)
Head & neck	344.2 (237.3)	78.4 (10.6)	27.8 (22.6)	10 (66.7)	1408.7 (403.6)	12 (80.0)	1503.1 (288.4)
Laryngology	277.5 (148.1)	75.3 (12.9)	28.6 (22.6)	10 (66.7)	1352.1 (632.9)	13 (86.7)	1574.3 (495.8)
Otology	227.9 (189.5)	68.5 (19.5)	51.7 (41.2)	9 (60.0)	1391.0 (668.8)	12 (80.0)	1596.0 (355.1)
Pediatrics	263.9 (161.5)	69.1 (23.9)	38.4 (32.1)	9 (60.0)	1243.2 (609.5)	12 (80.0)	1386.0 (328.6)
Pharmacology	195.1 (147.2)	73.3 (19.2)	53.1 (43.4)	12 (80.0)	1218.2 (515.6)	13 (86.7)	1360.1 (401.5)
Plastic & reconstructive	285.1 (183.5)	72.9 (21.7)	36.2 (28.6)	14 (93.3)	1451.9 (737.7)	13 (86.7)	1354.7 (471.3)
Rhinology	264.1 (186.9)	63.4 (26.6)	27.4 (19.6)	12 (80.0)	1282.7 (674.6)	13 (86.7)	1324.7 (448.8)
Sleep	213.1 (177.6)	70.3 (24.2)	45.5 (39.5)	11 (73.3)	1315.9 (694.6)	12 (80.0)	1511.2 (466.7)
Total	247.4 (176.6)	72.7 (19.3)	38.1 (33.4)	108 (72.0)	1305.5 (581.7)	122 (81.3)	1450.9 (402.4)

Abbreviations: SD, standard deviation

For standard GPT-4, mean question length (251 vs. 229 characters, *P* = 0.502), correct answer option length (33 vs. 50 characters, *P* = 0.016), and GPT-4 response length (1,204 vs. 1,565 characters, *P* < 0.001) varied between questions answered correctly and incorrectly. For custom GPT-4, mean question length (248 vs. 229 characters), correct answer option length (37 vs. 42 characters), and GPT-4 response length (1,460 vs. 1,449 characters) were similar between questions answered correctly and incorrectly.

## Discussion

Overall, our study demonstrated improved performance by standard and custom GPT-4, with more correct answers on ‘easy,’ longer, and plastic and reconstructive questions. Our study found that standard (72.0%) and custom GPT-4 (81.3%) demonstrated higher accuracy than GPT-3.5 on the same 150 questions included in our previous study (51.3%) and on similar Otolaryngology board-style questions (53%) [2, 3]. Custom GPT-4 outperformed otolaryngology trainees who averaged 72.7% accuracy. These findings align with recent studies showing GPT-4 outperforming GPT-3.5 on board-style questions for the United States Medical Licensing Examination, Plastic Surgery Inservice Training Examination, and National Board of Medical Examiners Surgery Subject Examination, demonstrating broad applicability of AI in medical education [4–6].

The higher accuracy demonstrated by Custom GPT-4 may be attributable to its instructions to specialize in Otolaryngology board-style questions, select one answer, and validate answers using the internet. Whereas custom GPT-4 always selected one answer, standard GPT-4 selected multiple or no answers 8.7% of the time. Custom models may enhance utilization of ChatGPT in medical education. There is the risk of intellectual dependency on AI and a perceived decrease in the need to learn complex pathophysiology. However, the present state of AI requires biomedical knowledge of diseases and critical thinking to appraise newly developed AI systems [7].

This study has several limitations including lack of repeated trials to account for variance in model output, because GPT-4 provides unique answers to each query. Questions with images were excluded because GPT-3.5 is limited to text input. The subject matter of our 150 board-style questions may not generalize to other fields. The functionality of GPT-4 may be limited in certain environments by the requirement of accessing GPT-4 with internet.

## Conclusion

Our study found performance improvements of GPT-4 over GPT-3.5 on Otolaryngology board-style questions. The additional capabilities of custom GPT-4 allowed us to create a model specializing in Otolaryngology-board style questions, which demonstrated higher accuracy than standard GPT-4. It is important to heed the risk of intellectual dependency on AI and to approach AI models with critical thinking and a background of knowledge. Future studies should explore benefits conferred to otolaryngology trainees utilizing AI for medical education. With the ability to interact with users, provide explanations, and adjust to user customizations, AI-based text models may continue to improve as tools for medical education.
